# The correlation between illness perception, pain intensity and quality of life in elderly with low back pain in Denmark: a cross-sectional study

**DOI:** 10.7717/peerj.14129

**Published:** 2022-10-14

**Authors:** Elisabeth Ginnerup-Nielsen, Mette Harreby, Robin Christensen, Henning Bliddal, Marius Henriksen

**Affiliations:** 1 Bispebjerg and Frederiksberg Hospital, The Parker Institute, Copenhagen, Denmark; 2 Department of Clinical Research, University of Southern Denmark, Odense University Hospital, Research Unit of Rheumatology, Odense, Denmark

**Keywords:** Low back pain, Illness perception, Quality of life, Pain management, Survey, Cross sectional study

## Abstract

**Background:**

Illness perception is related to management patterns and pain intensity, but among elderly with low back pain, this relation is unclear. The aims of this study were to analyse the associations between illness perception, pain intensity and health related quality of life in a group of elderly with low back pain and explore how different illness perception profiles would cluster and differ in terms of pain, quality of life and choice of management.

**Method:**

This was a cross-sectional survey based on a cohort of originally 640 Danish children. Of the 311 respondents in 2019, 69% reported low back pain within last year and were included. Associations between illness perceptions (Brief illness perception questionnaire), health related quality of life (EuroQol-5 Domain-3L) and low back pain intensity were assessed, and participants were clustered based on their perceptions using hierarchical and K-means cluster analysis. Cluster differences in pain, quality of life and use of pharmacological and non-pharmacological treatments were explored.

**Results:**

Among the 213 individuals with low back pain, 33% reported severe or fluctuating pain intensity. Higher pain intensity was associated with perceiving low back pain as a greater threat. Participants reporting fluctuating pain perceived their low back pain almost as threatening as participants reporting severe pain. Two clusters were identified. Cluster 1 reported lower quality of life (difference in medians: −0.176 (95% CI [−0.233–−0.119 ])) and was more likely to report severe or fluctuating pain (37.7% vs. 4.5% [*P* < 0.0001]) and to use pharmacological treatments than Cluster 2 (37.7% vs. 14.9% [*P* < 0.001]). No association was found between clusters concerning use of non-pharmacological treatments (*P* = 0.134).

**Conclusion:**

Based on illness perceptions, two clusters differing in pain intensity, quality of life and use of pharmacological treatments were identified. Targeting illness perceptions may be beneficial during rehabilitation or when guiding patients with low back pain in choice of management.

## Introduction

The lifetime prevalence of low back pain (LBP) is around 70–80%. People suffering from low back pain often report severely affected quality of life (Cedraschi et al., 2016; Dutmer et al., 2019; Ludwig et al., 2018) and in 2016, the Global Burden of Disease study (Vos et al., 2017) ranked LBP as the leading cause of years lived with disability. As the prevalence of LBP increases with higher age (Dionne, Dunn & Croft, 2006; Thomas et al., 2004) numbers will probably be rising in the coming years.

Several management strategies are being recommended for LBP (Corp et al., 2021; Qaseem et al., 2017b) with non-pharmacological treatments, such as exercise and self-care advice, being first choice. An understanding of the long term effects of these management strategies is, however, lacking (Hayden et al., 2019; Van Middelkoop et al., 2011). This could be due to unhelpful beliefs about treatments for LBP (Christe et al., 2021) or lack of adherence to rehabilitation (Beinart et al., 2013; Mailloux, Finno & Rainville, 2006).

Illness perceptions (IP) are the beliefs that one creates when faced with an illness. The concept of IP is based on the common-sense model with individuals expected to perceive the impact of their illness through five components: consequences (effects on life, including emotional representation) timeline (chronicity of illness), control/cure (controllability and potential effect of treatment), identity (associated symptoms) and cause. This understanding leads to a perception of the disease as either manageable or threatening and hereby influences the ability to cope with the disease (Leventhal et al., 1997).

Some studies have found IP components to be associated to LBP and quality of life (QoL) and to changes in these measures over time (Fors et al., 2022; Foster et al., 2008; Ünal et al., 2019), but studies exploring these relations in the elderly are few. As age-related differences in IP components have been found, with elderly being less emotionally affected by chronic illness (Leventhal, 1984), age related differences in how IP components associate with pain and QoL may be present as well. Hence, associations need to be confirmed among elderly with LBP.

Being related to coping, it appears likely that perceptions could influence choices of management, however, study results are ambiguous concerning this relation. Thus, while some studies have found that people perceiving their condition as a threat, are more likely to adhere to treatments (Frostholm et al., 2005; Ginnerup-Nielsen et al., 2021; Patel & Taylor, 2002). Bishop et al. (2017) found, in 324 patients receiving acupuncture for low back pain, that perceptions of LBP causing many severe symptoms (*e.g.*, LBP perceived as a threat) decreased the odds of attending all treatment sessions.

As the five components constituting illness perception (Leventhal et al., 1997) may impact on both clinical measures and treatment choices in different ways, using cluster analysis to identify subgroups of patients with similar perception traits seems appropriate and has also been recommended (Langenmaier et al., 2019; Viniol et al., 2013; Windgassen et al., 2018). Finding clusters of patients with common perceptions and treatment needs or preferences could potentially guide clinicians on how to advise patients concerning management of their LBP, eventually optimizing adherence to treatment.

Considering the relatively few studies exploring the relation between IP, pain and QoL, and the ambiguity concerning the impact of IP on choice of LBP treatment, the two aims of this study were to analyse the associations between IP, pain intensity and quality of life among a cohort of elderly citizens reporting LBP and to explore if different IP profiles exist in this cohort and how these profiles differ in terms of choice of management strategies, LBP intensity and QoL.

### Hypotheses

We hypothesised that IP, pain intensity and QoL would be associated, *i.e.,* people perceiving their LBP as a greater threat to them would report more pain and lower QoL and would tend to use more treatments and more often use pharmacological treatments for LBP (as opposed to non-pharmacological treatments).

## Materials & Methods

### Participants

This cross-sectional survey study is based on an inception cohort from the county of Elsinore, Denmark of 640 children (330 females and 310 males) who, in 1965, as 14-year-old pupils had their history of LBP registered by the local school doctor (Harreby et al., 1995). The follow-up study was conducted in September–October 2019. Participants now had a mean age of 68.5 (SD: 0.5) years.

### Online survey

The survey was adaptive based on how each respondent answered. As around 30 questions were added specifically to this survey (and not part of the original cohort survey), comprehension of the questions was evaluated on five citizens with LBP, by means of cognitive interviewing using the think-aloud technique (Willis, 2004). Afterwards, to test functionality and completeness of the questionnaire, all questions as well as information material were pilot tested in 25 citizens between 60–70 years with 60% having LBP.

Data were collected *via* REDCap, as previously described in Ginnerup-Nielsen et al. (2021). To minimize the risk of incorrect use and false (double) responses, the survey was sent through the public “Digital Post” system (electronic mailbox for letters from Danish authorities) administered by an online platform “e-Boks” (e Boks, 2021), linked to the individual’s personal identification number. Access to e-Boks is mandatory for people who do not have special needs. Around 90% of 60–70 year old Danes have access to e-Boks (Danmarks Statistik, 2017).

Participants were sent an e-letter, with a link to an online survey that was managed *via* REDCap (Research Electronic Data Capture) electronic data capture tools hosted at the capital region of Denmark. REDCap is a secure, web-based software platform designed for data collection in research studies (Harris et al., 2019; Harris et al., 2009). The letter included information about the study and questionnaire as well as the possibility to withdraw from the study at any time. Participants were encouraged to respond to the questionnaire whether they had LBP or not. All subjects gave informed consent when initiating the survey. In case of incomplete or non-response, two reminders were sent within six weeks.

### Ethics approval

The Regional Health Research Ethics Committee of the Capital Region of Denmark reviewed the outline of this cohort study. The committee deemed the study exempt from approval (Reference no.: H-20012468) as it was only based on questionnaires. Such studies can be implemented without permission from the Health Research Ethics Committee according to Danish legislation (Committee Act §1, paragraph 1).

### Variables and outcome measures

The questionnaire included a maximum of 52 questions for people reporting no LBP and 105 questions for people reporting LBP based on the two initial triage questions: “Have you ever had low back pain”—With response options: “yes” and “no” and “How many days have you had low back pain within the last 12 months?” with response options: “0 days”, “1–7 days”, “8-30 days”, “more than 30 days, but not daily” or “daily”. Analyses in this study are based on people who reported LBP at least 1–7 days within the last year.

The rest of the questions concerned LBP intensity—with response options: “mild”, “moderate”, “severe” and “fluctuating” and use of medications and treatments for LBP. It furthermore concerned earlier back pain related illnesses and examinations or surgeries as well as IP (related to LBP), health related QoL, musculoskeletal health, fitness and physical function, lifestyle, and demographics.

### The brief illness perception questionnaire

The Danish version of The Brief illness perception questionnaire (B-IPQ) was used to assess illness perceptions (Broadbent et al., 2006a). The B-IPQ is a generic questionnaire developed as a short version of the original 84-item revised illness perception questionnaire (IPQ-R) (Moss-Morris et al., 2002) and assesses illness perceptions based on the five dimensions: Identity, Cause, Timeline, Consequences and Cure-Control (Broadbent et al., 2006a).

The B-IPQ contains nine items. The first eight items are scored on a numeric rating scale (1–10) with 1 being no perceived threat (*e.g.*, no affect at all) in items 1, 2, 5, 6 and 8 and highest perceived threat (*e.g.*, no control at all) in items 3, 4 and 7. Item 9 is a free text field in which the respondent can formulate their beliefs about their condition. Both the English and Danish versions of the B-IPQ are available online (Broadbent et al., 2006b) and the wording of the B-IPQ version used in this study is presented in Table 1. We only present analyses based on the questions 1-8.

**Table 1:** Verbatim description of the Brief illness perception questionnaire.

**Illness perception domain**	**Question**	**Response anchors**
Consequences	How much does your low back pain affect your life?	No affect at all
		Severely affects my life
Timeline	How long do you think your low back pain will continue?	A very short time
		Forever
Personal control	How much control do you feel you have over your low back pain?	Absolutely no control
		Extreme amount of control
Treatment control	How much do you think treatment can help your low back pain?	Not at all
		Extremely helpful
Identity	How much do you experience symptoms from your low back pain?	No symptoms at all
		Many severe symptoms
Concern	How concerned are you about your low back pain?	Not at all concerned
		Extremely concerned
Coherence	How well do you feel you understand your low back pain?	Don’t understand at all
		Understand very clearly
Emotional representation	How much does your low back pain affect you emotionally? (*e.g.*, does it make you angry, scared, upset or depressed?	Not at all affected emotionally
		Extremely affected emotionally

We replaced the word “illness” with “low back pain” in all items which is recommended when using the B-IPQ in specific conditions (Broadbent et al., 2006a). Furthermore, we changed the wording in item 4 from “How much do you think *your* treatment can help your low back pain?” to “How much do you think treatment can help your low back pain?” as our respondents were not necessarily being treated for their LBP.

Although an overall score can be calculated from the B-IPQ questionnaire (Løchting et al., 2013), items are usually presented separately. Accordingly, analyses in this study were based on each item score.

### EQ-5D (3L) and EQ-VAS

We used the EuroQoL 5 Domain (EQ-5D) questionnaire with three response options to assess health related QoL. The EQ-5D is a standardised measure of health status that provides a simple, generic health measure. It is simple to use and has been validated in a wide range of health conditions and languages (Rabin & Charro, 2001). The EQ-5D covers questions within five dimensions: mobility, self-care, usual activities, pain/discomfort, and anxiety/depression. Response options are given *via* three Likert boxes ranging from being in perfect health (*e.g.*, I have no problems in walking about) to being seriously affected (*e.g.*, I am confined to bed). From the responses, an EQ-5D index is calculated ranging from −0.624 (worst rated health) to 1.000 (best rated health) (Lauritsen, 2007).

The EQ-5D also contains an EQ visual analogue scale (0-100) called EQ VAS. The scale is used to record the respondent’s self-rated health with endpoints labelled “the best health you can imagine” and “the worst health you can imagine” (EuroQol Group, 1990). In 2009 the EQ-5D was translated into Danish and a Danish valuation set for reference was developed (Wittrup-Jensen et al., 2009).

### Self-management strategies

Self-management strategies included the use of different predefined types of non-pharmacological or pharmacological treatments for LBP the last 12 months. Users were described based on treatment type: they could be users of pharmacological treatments and users of non-pharmacological treatments. Furthermore, the number of users of any kind of treatment (at least one type of either non-pharmacological or pharmacological treatment) was calculated.

A user of both pharmacological and non- pharmacological treatments was included in both groups.

Use of pharmacological treatments within the last 12 months was defined by a positive response to the question: “Have you taken painkillers because of low back pain within the last 12 months (Tablets, capsules, suppositories, oral solution, injections)?”.

Use of non-pharmacological treatments included receiving either: physiotherapy and/or chiropractic’s specifically for LBP within the last 12 months and/or doing low back pain specific exercise at least weekly.

As a reference from the general practitioner (GP) is needed to access physiotherapy or receive prescription drugs we decided not to include visit to the GP as a self-management strategy.

### Statistical analyses

This study was designed as a descriptive cross-sectional study.

The included population was described regarding to sex, age, LBP intensity, disease management (pharmacological/non-pharmacological), B-IPQ scores and EQ-5D-scores. As data generally did not follow a normal distribution, medians, and interquartile ranges (IQR) were presented.

Correlations between IP (B-IPQ) and QoL (EQ-5D and EQ-VAS) were explored using Spearman’s correlation. Given that there is no consistent interpretation of the correlation coefficient, interpretation was based on rule of thumb: Correlations below 0.3, between 0.3 to 0.6 and greater than 0.6 were considered low, moderate, and high, respectively (Andresen, 2000; Overholser & Sowinski, 2008).

Associations between pain intensity and IP were explored *via* Kruskal-Wallis one way analysis of variance (ANOVA) and presented in boxplots.

### Cluster analysis

To investigate how eventual IP based profiles would differ in terms of LBP intensity, QoL and use of treatments, a cluster analysis (CA) based on the B-IPQ-item scores was performed and the distribution of users of pharmacological or non-pharmacological treatments as well as the levels of pain intensity and QoL (EQ-5D and EQ-VAS) in the resulting clusters were explored. The CA was performed using the 2-step procedure recommended by Clatworthy et al. (2007) and described in Ginnerup-Nielsen et al. (2021). First, we performed a hierarchical analysis using Ward’s method with squared Euclidean Distance based on a reformed agglomeration schedule to determine the optimal number of clusters, then, we used the centroids and the number of clusters from the analysis as a starting point in a K-means CA.

*χ* test (with Fisher’s exact test for expected counts <5) was used to assess differences in cluster distributions of males/females and users of pharmacological/non-pharmacological treatments. Kruskal-Wallis test for non-parametric independent samples was used to assess cluster differences in age, QoL and pain intensity.

All analyses were performed in SPSS (version 3.3). All *P*-values and 95% confidence intervals were two-sided. Statistical significance was set at an *α* level of 0.05.

Statistical tests were reported with *P*-values for standard hypothesis tests and claims of statistical significance were only intended for exploratory purposes.

## Results

**Figure 1:**
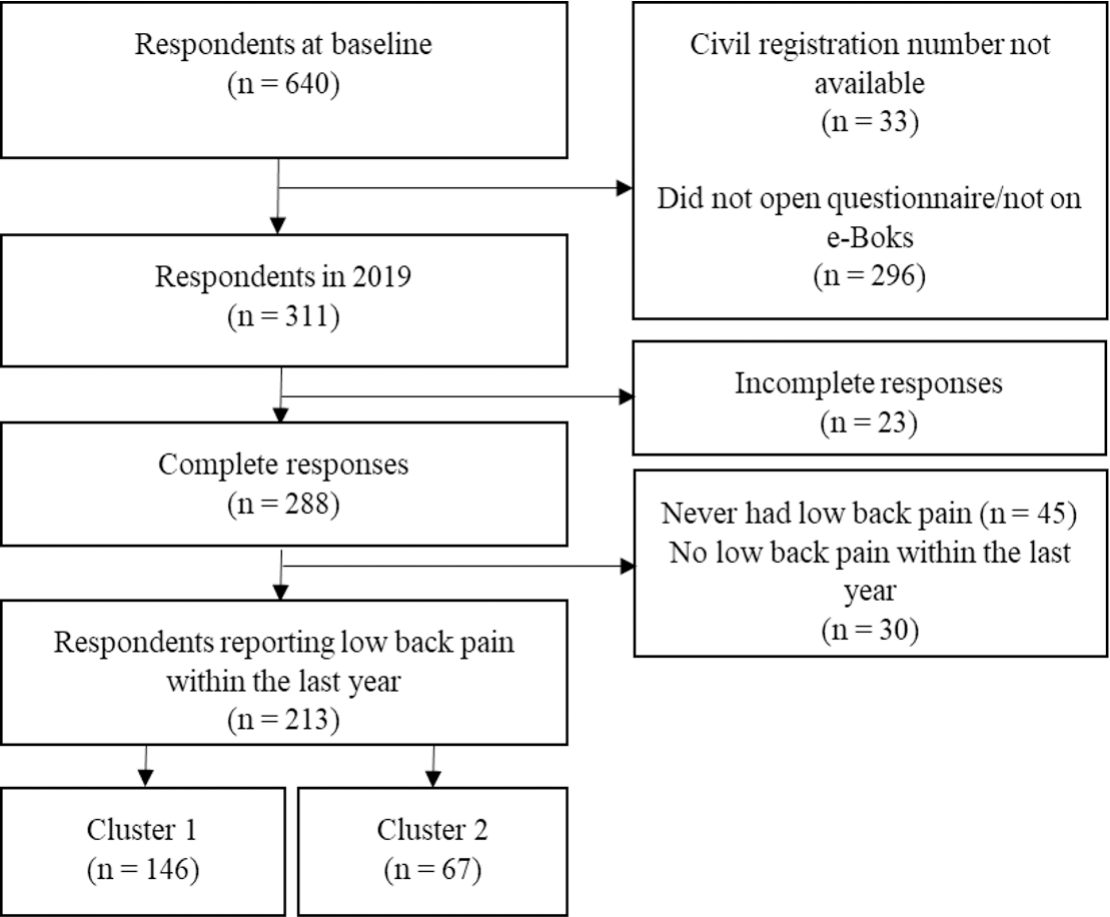
Flowchart of respondents flow.

Among a total of 640 participants in the initial 1965 Elsinore Cohort, we discarded 33 (5.2%) in the present 2019 survey due to incorrect civil registration numbers. Hence, questionnaires were sent to 607 participants of which 311 (51.2%) responded, with 288 (47.4%) complete responses and 23 (3.8%) incomplete responses. 258 (42.5%) had experienced LBP throughout their lives and 213 (35.1%) reported having had LBP within the last year. The flow of participants from the full cohort to the clusters found in this project is shown in Fig. 1.

This study is based on respondents with full responses who reported LBP within the last year (*N* = 213). Before any study related activities were undertaken, a protocol was published (protocol number: NCT03940456).

Of the 213 included respondents, 118 (55.4%) were women, 137 (64%) reported use of any kind of treatment with 65 (30.5%) having used at least one type of pharmacological treatment and 124 (58.2%) at least one type of non-pharmacological treatment within the last 12 months.

Eighty-six (40.4%) had mild LBP, 69 (32.4%) had moderate LBP, 12 (5.5%) had severe LBP and 46 (21.6%) had fluctuating LBP. Eighty-nine (41.8%) reported pain episodes between 1–7 days and 38 (17.8%) reported daily pain. A summary of participant characteristics is presented in Table 2.

### Brief illness perception scores

At item level, the lowest scores were seen on item 8 (emotional representation): “How much does your low back pain affect you emotionally?” with a median of 2 (IQR 1.0−3.5) and the highest scores were seen on item 2 (Timeline): “How long do you think your low back pain will continue?” with a median of 9.0 (IQR 3.5-10), indicating that, despite heterogenic responses, participants generally expected their LBP to continue for very long but were not considerably emotionally affected by it.

**Table 2:** Participant characteristics. Values are median (IQR), unless otherwise stated. EQ-5D Index: EuroQol-5 Domain and EQVAS: higher scores denote better quality of life. Brief-IPQ (B-IPQ): Brief illness perception questionnaire, higher scores on item 1, 2, 5, 6, 8 and overall item score denote a more threatening view of the illness, while higher scores on item 3, 4 and 7 denote a less threatening view of the illness.

**Variables**	**N (213)**	**N (%)/Median (IQR)**
**Demographics**		
Women N (%)	118 (55.4%)	
Age years, median (IQR)	213	69 (68–69)
**Frequency of LBP last year n (%)**		
1–7 days	213	89 (41.8%)
8–30 days	213	45 (21.1%)
More than thirty days, but not daily	213	41 (19.2%)
Daily	213	38 (17.8%)
**LBP intensity n (%)**		
Mild	213	86 (40.4%)
Moderate	213	69 (32.4%)
Severe	213	12 (5.6%)
Fluctuating severity	213	46 (21.6%)
**Non-pharmacological treatments for LBP last 12 months, n (%) (at least one type)**	213	124 (58.2%)
Physiotherapy	124	38 (30.6%)
Chiropractor	124	31 (25%)
Exercise (at least once a week)	124	101 (81.5%)
Other	124	4 (3.2%)
**Pharmalogical treatments for LBP last year 12 months, n (%)**	213	65 (30.5%)
Daily use of pharmacological treatments for LBP, n (%)	213	21 (9.9%)
**User of any treatment (non-pharmacological or pharmacological) (n, %)**	213	137 (64.3%)
**Health related quality of life, median (IQR)**		
EQ-5D Index	213	0.824 (0.773–1.000)
EQ-VAS	213	80 (66–88.5)
**B-IPQ items 1–8, score 1–10 (median, IQR)**		
Item 1 - consequences	213	3.0 (2.0–7.5)
Item 2 - timeline	213	9.0 (3.5–10)
Item 3 - personal control	213	7.0 (5.0–9.0)
Item 4 - treatment control	213	5.0 (3.0–8.0)
Item 5 - identity	213	4.0 (2.0–5.0)
Item 6 - concern	213	3.0 (1.0–5.0)
Item 7 - coherence	213	8.0 (5.0–10)
Item 8 - emotions	213	2.0 (1.0–3.5)

On the item 3 (personal control): “How much control do you feel you have over your low back pain?”, participants scored a median of 7.0 (IQR 5.0−9.0) while the median score on item 4 (treatment control): “How much do you think treatment can help your low back pain?” was 5 (IQR 3.0−8.0), indicating that participants generally felt in control of their pain, but did not necessarily trust in treatment to have an effect although scores also demonstrated heterogeneity between respondents (Table 2).

### Quality of life (EQ-5D and EQ-VAS)

The median EQ-5D score was 0.824 (IQR: 0.773−1.000) while the median EQ-VAS was 80 (IQR: 66–88.5) indicating that participants’ QoL was generally mildly affected according to age adjusted Danish population norms (Sørensen et al., 2009).

### Correlations between illness perception items

Analyses of the interrelationships between the B-IPQ items showed moderate to high statistically significant correlation coefficients between the perceived severity of LBP symptoms (item 5, Identity) and consequences of LBP (item 1, Consequences) (r: 0.76). Hence, participants with more severe symptoms perceived their LBP to have more consequences for their lives. Furthermore, participants who were more concerned about their LBP (item 6, Concern) were more emotionally affected (item 8, Emotions) (r: 0.72), they also perceived more consequences to their LBP (item 1, Consequences) (r: 0.69) and had lower QoL (EQ-5D) (r: −0.62).

Moderate statistically significant correlations with coefficients ranging from −0.32 to 0.59 were seen for the rest of the B-IPQ items, except from a low correlation of (r: 0.15) between item 2 (Timeline) and item 7 (Coherence) and (r: 0.23), and item 2 (Timeline) and item 8 (Emotions). Generally, low and mostly non-statistically significant correlations were found between item 4 (Treatment control) and all other B-IPQ items, indicating that participants’ perceptions about possible effects of treatment were not related to other perceptions (Table 3).

**Table 3:** Correlations between Brief illness perception questionnaire items and health related outcome measures.

** **	**B-IPQ item 1 Consequences**	**B-IPQ item 2 Timeline**	**B-IPQ item 3 Personal control**	**B-IPQ item 4 Treatment control**	**B-IPQ item 5 Identity**	**B-IPQ item 6 Concern**	**B-IPQ item 7 Coherence**	**B-IPQ item 8 Emotions**	**EQ-5D-Index**	**EQ-VAS**
**B-IPQ item 1 Consequences**	** **	.576 (*p* = 0.01)	−.550 (*p* = 0.01)	0.103 (*P* = 0.14)	.756 (*p* = 0.01)	.686 (*p* = 0.01)	−.320 (*p* = 0.01)	.586 (*p* = 0.01)	−.623 (*p* = 0.01)	−.564 (*p* = 0.01)
**B-IPQ item 2 Timeline**	** **		−.438 (*p* = 0.01)	0.090 (*P* = 0.19)	.509 (*p* = 0.01)	.386 (*p* = 0.01)	−.151 (*P* = 0.05)	.297 (*p* = 0.01)	−.369 (*p* = 0.01)	−.359 (*p* = 0.01)
**B-IPQ item 3 Personal control**	** **			−0.100 (*P* = 0.15)	−.462 (*p* = 0.01)	−.525 (*p* = 0.01)	.464 (*p* = 0.01)	−.480 (*p* = 0.01)	.442 (*p* = 0.01)	.534 (*p* = 0.01)
**B-IPQ item 4 Treatment control**	** **				.183 (*p* = 0.01)	.163 (*P* = 0.05)	−0.034 (*P* = 0.62)	.147 (*P* = 0.05)	−0.088 (*P* = 0.20)	0.025 (*P* = 0.72)
**B-IPQ item 5 Identity**	** **					.670 (*p* = 0.01)	−.281 (*p* = 0.01)	.576 (*p* = 0.01)	−.568 (*p* = 0.01)	−.444 (*p* = 0.01)
**B-IPQ item 6 Concern**	** **						−.440 (*p* = 0.01)	.718 (*p* = 0.01)	−.539 (*p* = 0.01)	−.456 (*p* = 0.01)
**B-IPQ item 7 Coherence**	** **							−.367 (*p* = 0.01)	.269 (*p* = 0.01)	.354 (*p* = 0.01)
**B-IPQ item 8 Emotions**	** **								−.474 (*p* = 0.01)	−.363 (*p* = 0.01)
**EQ-5D-Index**	** **									.599 (*p* = 0.01)
**EQ-VAS**	** **									

**Notes.**

** Correlation is significant at the 0.01 level (2-tailed).

* Correlation is significant at the 0.05 level (2-tailed).

### Correlations between illness perception and EQ-5D

High, statistically significant correlations were found between the B-IPQ item 1 (Consequences) and the EQ-5D (r: −0.62). Low to no correlation was seen between B-IPQ item 4 (Treatment control) and any other health related outcome measure and low to moderate correlations were found between all B-IPQ items and EQ-5D and EQ-VAS (rs: 0.27 to −0.53). Correlations indicated that the more threatening participants perceived their LBP, the lower QoL they reported (Table 3).

### Associations between illness perception and pain intensity

**Figure 2:**
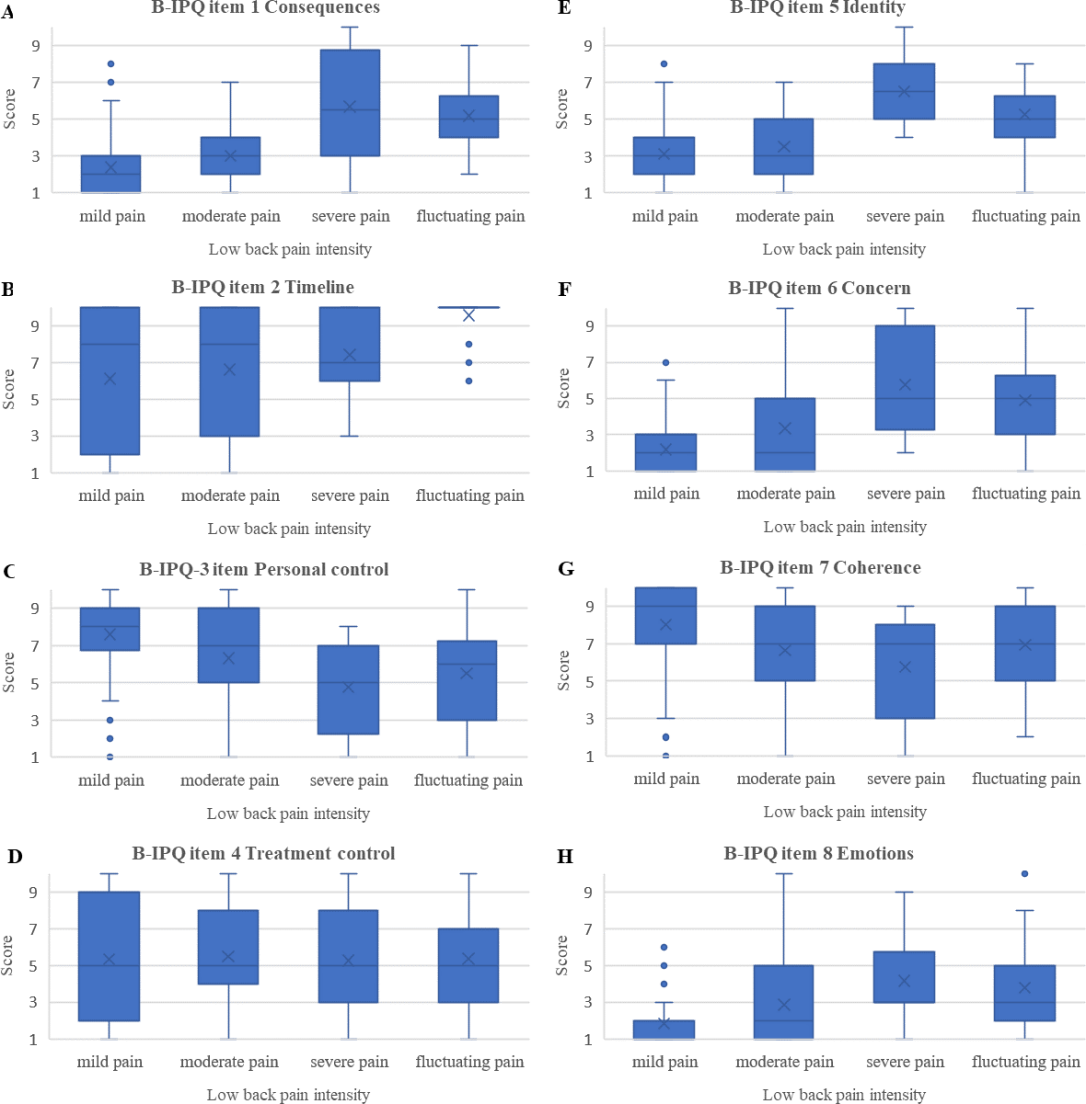
Boxplots of associations between pain intensity and Brief Illness perception questionnaire scores (median, upper, and lower quartiles and outliers). (A) B-IPQ item 1 consequences. (B) B-IPQ item 2 timeline. (C) B-IPQ item 3 personal control. (D) B-IPQ item 4 treatment control. (E) B-IPQ item 5 identity. (F) B-IPQ item 6 concern. (G) B-IPQ item 7 coherence. (H) B-IPQ item 8 emotions.

We found all IP items, except from item 4 (treatment control) (*P* = 0.06), to be associated with pain levels (*P* < 0.01), with higher pain levels being associated with perceiving LBP as a greater threat. Participants reporting fluctuating pain patterns perceived their LBP almost as threatening as participants reporting severe pain (Fig. 2).

### Cluster analysis based on illness perception scores

Based on the change between coefficients in the agglomeration schedule from the hierarchical CA and the subsequent K-Means analysis, we found that two illness perception clusters would be the optimal clustering solution.

In the two-cluster solution we found statistically significant cluster differences in all B-IPQ item scores, except item 4, as well as in EQ-5D and EQ-VAS scores (Table 4).

**Table 4:** Characteristics of clusters and comparisons between groups. Values are median (IQR), unless otherwise stated. *Statistical significance accepted at *P* < 0.05. Fischers exact test was used when expected counts were <5. EQ5D: EuroQol-5 Domain and EQ-VAS: higher scores denote better quality of life. Brief-IPQ (B-IPQ): Brief illness perception questionnaire, higher scores on item 1, 2, 5, 6, 8 and overall score denote a more threatening view of the illness, while higher scores on item 3, 4 and 7 denote a less threatening view of the illness.

	**Cluster 1** **(*N* = 146)**	**Cluster 2 (*N* = 67)**	**Difference**	
			**Median (95% CI)**	*P*-value^*^
**Demographics**				
Women N (%)	83 (56.8%)	35 (52.2%)		0.53
Age years, median (IQR)	69 (68–69)	68 (68–69)		0.37
**Frequency of LBP last year, n (%)**				*P* < 0.0001
1–7 days	41 (28.1%)	48 (71.6%)	n.a.	
8–30 days	31 (21.2%)	14 (20.9%)	n.a.	
More than thirty days, but not daily	36 (24.7%)	5 (7.5%)	n.a.	
Daily	38 (26.0%)	0	n.a.	
**LBP intensity n (%)**				*P* < 0.0001
Mild	48 (32.9%)	38 (56.7%)	n.a.	
Moderate	43 (29.5%)	26 (38.81)	n.a.	
Severe	9 (6.2%)	3 (4.5%)	n.a.	
Fluctuating severity	46 (31.5%)	0	n.a.	** **
**Treatments n (%)**				
Pharmacological treatments	55 (37.7%)	10 (14.9%)	n.a.	*P* < 0.001
Non-pharmacological treatments	90 (61.6%)	34 (50.7%)	n.a.	0.134
Any treatments#	99 (67.8%)	38 (56.7%)	n.a.	0.117
Daily use of pharmacological treatments LBP, n (%)	20 (13.7%)	1 (1.5%)	n.a.	0.148
**Health related quality of life, (median, IQR)**				
EQ-5D-Index	0.824 (0.746–0.824)	1.000 (0.824–1.000)	−0.176 (−0.233 to −0.119)	0.0001
EQ-VAS	75.00 (60.0–82.3)	86.00 (80.0–90.0)	−11 (−14.42 to −7.58)	0.0001
**B-IPQ items 1–8, score 1–10, (median, IQR)**				
Item 1 - consequences	4.0 (3.0–5.0)	2.0 (1.0–2.0)	2 (1.7 to 2.3)	0.0001
Item 2 - timeline	10.0 (8.8–10.0)	2 (1.0–3.0)	8 (7.2 to 8.8)	0.0001
Item 3 - personal control	6.0 (4.0–8.0)	9.0 (8.0–10.0)	−3 (−3.6 to −2.4)	0.0001
Item 4 - treatment control	5.0 (3.8–8.0)	5.0 (2.0–8.0)	0	0.076
Item 5 - identity	5.0 (3.0–6.0)	2.0 (1.0–3.0)	3 (2.4 to 3.6)	0.0001
Item 6 - concern	3.0 (2.0–5.0)	1.0 (1.0–2.0)	2 (1.5 to 2.6)	0.0001
Item 7 - coherence	8.0 (5.0–9.0)	9.0 (7.0–10.0)	−1 (−1.7 to −0.3)	0.005
Item 8 - emotions	2.0 (1.0–5.0)	1.0 (1.0–2.0)	1 (0.7 to 1.3)	0.0001

**Notes.**

* Statistical significance accepted at *P* < 0.05.

Cluster 1 contained 146 participants while Cluster 2 contained 67 participants. In Cluster 1, 41 (28.1%) had experienced LBP 1-7 days the last year while 38 (26.0%) had daily pain, 48 (32.9%) characterized their pain as mild, while 9 (6.2%) characterized their pain as severe. Cluster 1 reported lower quality of life than Cluster 2 with a difference between medians of −0.176 (95% CI [−0.233–−0.119]) on the EQ-5D.

Cluster 1 generally perceived LBP as a greater threat than Cluster 2. Cluster 1 was more concerned (item 6) (difference between medians: 2 (95% CI [1.45–2.55])) and perceived LBP to cause more symptoms (item 5) and have more consequences (item 1) than Cluster 2 with differences between medians of respectively: 3 (95% CI [2.37–3.63]) and 2 (95% CI [1.66–2.34]). Respondents in Cluster 1 also expected their LBP to last much longer than respondents in Cluster 2 (item 2) (difference between medians: 8 (95% CI [7.18–8.82])) and perceived themselves to be in less control of their pain than respondents in Cluster 2 (difference between medians: −3 (95% CI [−3.63–−2.37])). No statistically significant difference was found between clusters concerning their confidence in the effect of treatment for their LBP (item 4).

In Cluster 2, 48 (71.6%) had experienced LBP 1-7 days the last year while none reported daily pain, 38 (56.7%) characterized their pain as mild while 3 (4.5%) characterized their pain as severe. Hence, the population in Cluster 2 had experienced fewer days with LBP within last year and markedly fewer respondents reported severe or fluctuating pain patterns than in Cluster 1.

There was a statistically significant difference between clusters concerning use of pharmacological treatments, as 55 (37.7%) in Cluster 1 had used pharmacological treatment within the last 12 months and 20 (13.7%) reported daily use. In Cluster 2, 10 (14.9%) had taken pharmacological treatment within the last 12 months (*P* = 0.001) and only one (1.5%) reported daily use. No statistically significant difference was found between clusters in the use of non-pharmacological treatments or in the proportions of users of any kind of treatment. The proportions of women in the 2 clusters were 83 (56.8%) in Cluster 1 and 35 (52.2%) in Cluster 2 (Table 4).

## Discussion

In this cross-sectional study, based on 213 elderly reporting LBP within the last year, higher pain intensity was associated with perceiving LBP as a greater threat, with fluctuating pain patterns being perceived almost as threatening as severe pain.

We identified two IP-based cluster analysis with respondents in Cluster 1 perceiving their low back pain as a greater threat than respondents in Cluster 2. More respondents in Cluster 1 than in Cluster 2 reported severe or fluctuating LBP as well as daily pain. Furthermore, respondents in Cluster 1 were more likely to use pharmacological treatments, while no cluster difference was seen in the use of non-pharmacological treatments.

Overall, our respondents perceived their LBP as a minor threat and reported a better QoL than other similar studies (Andrew et al., 2014; Leadley et al., 2014; Løchting et al., 2013), probably because our study population was not necessarily being LBP patients. Respondents reporting fluctuating LBP perceived their pain almost as threatening as participants reporting severe pain, indicating that an unpredictable pain pattern is perceived as a great threat to the individual.

We found more threatening views of LBP to be associated with poorer QoL which has been found in other studies as well (Foster et al., 2008; Heyduck, Meffert & Glattacker, 2014; Løchting et al., 2013; Ünal et al., 2019). Furthermore, we found predominantly moderate to high inter-correlations between B-IPQ items except from the item 4 “Treatment Control” where no correlation was found which is also a pattern found in previous research within LBP (Løchting et al., 2013; Van Oort, Schröder & French, 2011). Despite this lack of correlation, many of our respondents reported regular exercise for their LBP which could suggest that our respondents do not consider regular exercise as an actual “LBP treatment”, but as a pain preventive measure.

In our CA, participants in Cluster 1, who, compared to participants in Cluster 2, perceived more symptoms to their LBP and perceived it to be more chronic with less personal control, were more likely to use pharmacological treatments than participants in Cluster 2.

In a recent cluster analysis of 1343 patients reporting LBP (Morton, De Bruin & Macfarlane, 2021), four clusters were identified: (1) a low threat cluster, (2) a high threat cluster, (3) a moderate threat–high treatment control cluster and (4) a moderate threat–low personal control cluster. Relative to the low threat cluster, the cluster perceiving LBP as a high threat was more likely to having contacted a general practitioner and the cluster perceiving LBP as a moderate threat with high confidence in the effect of treatment was more likely to having consulted a physical therapist.

In another cluster analysis of 117 patients taking hypertension medication (Hsiao, Chang & Chen, 2012), high perceived treatment control and personal control was found to be related to better treatment adherence.

We found no difference between clusters on the treatment control item which could again be due to the fact that our respondents were not necessarily being LBP patients or that they were relatively homogenous as all respondents were in the same age, as opposed to the populations in the above-mentioned studies with lower mean age and a greater age range. It seems likely that elderly, who may have experienced pain (illness) for a longer time, have less confidence in the effect of treatment regardless of pain levels.

We found item 2 (timeline) to differ markedly between clusters with Cluster 1 believing that LBP would last for a very long time. Perceptions of chronicity has, along with low perceived personal control, been found to predict poor QoL, higher pain levels and lower functional level over time in LBP patients (Fors et al., 2022; Foster et al., 2008), however, the relation between perception of chronicity and choice of treatments is unclear.

Conflicting results concerning the association between IP and illness management could be attributable to differences in treatment types or patient populations. As people will tend to seek common-sense between their illness perceptions and choice of treatments, two people with similar LBP related problems may perceive the necessity of a treatment very differently (Cameron & Leventhal, 2003). As an example, performing pain preventive exercises may seem relevant to a person with a predictable pain pattern, but irrelevant to someone with fluctuating unpredictable pain episodes or pain that has been chronic, and constant for years. Furthermore, there may also be disease related differences in how people perceive the importance of being in control of an illness; as exercise and self-management are some of the main recommended management strategies for low back pain, a relatively high degree of personal involvement may be required from the patient (as opposed to a disorder that is treated by medication). In this case, believing that the illness can be controlled by a treatment (treatment control item) may be essential for adherence.

In our study, although not necessarily being patients, respondents in Cluster 1, compared to Cluster 2, perceived their LBP to be more chronic. They also reported less personal control, more perceived symptoms, higher pain levels, lower quality of life and more use of pharmacological treatments. Targeting perceptions in this group could be relevant during therapy and both general practitioners, guiding patients in choices of treatments and therapists working in rehabilitation could gain from knowledge on how illness perception may relate to pain, QoL and treatment preferences.

This study has some limitations. First, as this is a cross-sectional study, the relationships we found between illness perceptions, management patterns and health related outcome measures were not necessarily causal. Our study population was based on an inception cohort from a relatively small area in Denmark, limiting generalizability. Further, we defined “management of low back pain” as the use of different predefined treatment strategies. We based our definitions on general recommendations on LBP management (Corp et al., 2021; Qaseem et al., 2017a), still, we may not have included all relevant treatments. Furthermore, we defined users as either: users of “non-pharmacological treatments”, pharmacological treatments” or “any kind of treatment”. It would also have been relevant to include a group of “users of both treatment types”.

Finally, due to constraints on the number of questions, we did not include LBP specific functional measures although this would have added useful information to the study.

The strengths of this study include a relatively large sample size and a good response rate. An electronic survey questionnaire applying branching was used, ensuring that all questions were presented and collected correctly. Furthermore, the association between illness perception and LBP intensity was documented both *via* ANOVA and cluster analysis.

## Conclusion

As hypothesised, among elderly citizens with LBP, perceiving LBP as a great threat was associated with higher pain levels and lower QoL but, in contrast to our hypotheses, IP was only associated with the use of pharmacological treatments but not with treatment use in general or with the use of non-pharmacological treatments. One third of our population reported severe or fluctuating pain and perceived their LBP as a threat. Including these perceptions when guiding patients in choice of management and during therapy may optimize treatment adherence and reduce pain.

##  Supplemental Information

10.7717/peerj.14129/supp-1 Supplemental Information 1 2019 cross sectional analysis of the Elsinore Cohort

10.7717/peerj.14129/supp-2 Supplemental Information 2 Strobe checklist

## Additional Information and Declarations

### Data Availability

The following information was supplied regarding data availability: The raw data is available as a Supplemental File.

## References

[ref-1] Andresen EM (2000). Criteria for assessing the tools of disability outcomes research. Archives of Physical Medicine and Rehabilitation.

[ref-2] Andrew R, Derry S, Taylor RS, Straube S, Phillips CJ (2014). The costs and consequences of adequately managed chronic non-cancer pain and chronic neuropathic pain. Pain Practice.

[ref-3] Beinart NA, Goodchild CE, Weinman JA, Ayis S, Godfrey EL (2013). Individual and intervention-related factors associated with adherence to home exercise in chronic low back pain: a systematic review. The Spine Journal.

[ref-4] Bishop FL, Yardley L, Cooper C, Little P, Lewith G (2017). Predicting adherence to acupuncture appointments for low back pain: a prospective observational study. BMC Complementary and Alternative Medicine.

[ref-5] Broadbent E, Petrie JK, Main J, Weinman J (2006a). The brief illness perception questionnaire. Journal of Psychosomatic Research.

[ref-6] Broadbent E, Petrie KJ, Main J, Weinman J (2006b). The Brief illness perception questionniare. https://ipq.h.uib.no/pdf/.

[ref-7] Cameron LD, Leventhal H (2003). The self-regulation of health and illness behaviour.

[ref-8] Cedraschi C, Luthy C, Allaz A-F, Herrmann F, Ludwig C (2016). Low back pain and health-related quality of life in community-dwelling older adults. European Spine Journal.

[ref-9] Christe G, Pizzolato V, Meyer M, Nzamba J, Pichonnaz C (2021). Unhelpful beliefs and attitudes about low back pain in the general population: a cross-sectional survey. Musculoskeletal Science and Practice.

[ref-10] Clatworthy J, Hankins M, Buick D, Weinman J, Horne R (2007). Cluster analysis in illness perception research: a Monte Carlo study to identify the most appropriate method. Psychology and Health.

[ref-11] Corp N, Mansell G, Stynes S, Wynne-Jones G, Morsø L, Hill JC, van der Windt DA (2021). Evidence-based treatment recommendations for neck and low back pain across Europe: a systematic review of guidelines. European Journal of Pain.

[ref-12] Danmarks Statistik (2017). Statistisk Årbog. https://www.dst.dk/da/Statistik/Publikationer/VisPub?cid=22259121.

[ref-13] Dionne CE, Dunn KM, Croft PR (2006). Does back pain prevalence really decrease with increasing age? A systematic review. Age and Ageing.

[ref-14] Dutmer AL, Preuper HRS, Soer R, Brouwer S, Bültmann U, Dijkstra PU, Coppes MH, Stegeman P, Buskens E, Van Asselt AD (2019). Personal and societal impact of low back pain: the Groningen spine cohort. Spine.

[ref-15] e Boks (2021). https://www.e-boks.com/danmark/en.

[ref-16] EuroQol Group (1990). EuroQol-a new facility for the measurement of health-related quality of life. Health Policy.

[ref-17] Fors M, Öberg B, Enthoven P, Schröder K, Abbott A (2022). The association between patients’ illness perceptions and longitudinal clinical outcome in patients with low back pain. Pain Reports.

[ref-18] Foster NE, Bishop A, Thomas E, Main C, Horne R, Weinman J, Hay E (2008). Illness perceptions of low back pain patients in primary care: what are they, do they change and are they associated with outcome?. Pain.

[ref-19] Frostholm L, Fink P, Christensen KS, Toft T, Oernboel E, Olesen F, Weinman J (2005). The patients’ illness perceptions and the use of primary health care. Psychosomatic Medicine.

[ref-20] Ginnerup-Nielsen E, Christensen R, Heitmann BL, Altman RD, March L, Woolf A, Bliddal H, Henriksen M (2021). Estimating the prevalence of knee pain and the association between illness perception profiles and self-management strategies in the frederiksberg cohort of elderly individuals with knee pain: a cross-sectional study. Journal of Clinical Medicine.

[ref-21] Harreby M, Neergaard K, Hesselsøe G, Kjer J (1995). Are radiologic changes in the thoracic and lumbar spine of adolescents risk factors for low back pain in adults? A 25-year prospective cohort study of 640 school children. Spine.

[ref-22] Harris PA, Taylor R, Minor BL, Elliott V, Fernandez M, O’Neal L, McLeod L, Delacqua G, Delacqua F, Kirby J (2019). The REDCap consortium: building an international community of software platform partners. Journal of Biomedical Informatics.

[ref-23] Harris PA, Taylor R, Thielke R, Payne J, Gonzalez N, Conde JG (2009). Research electronic data capture (REDCap)—a metadata-driven methodology and workflow process for providing translational research informatics support. Journal of Biomedical Informatics.

[ref-24] Hayden JA, Wilson MN, Riley RD, Iles R, Pincus T, Ogilvie R (2019). Individual recovery expectations and prognosis of outcomes in non-specific low back pain: prognostic factor review. Cochrane Database of Systematic Reviews.

[ref-25] Heyduck K, Meffert C, Glattacker M (2014). Illness and treatment perceptions of patients with chronic low back pain: characteristics and relation to individual, disease and interaction variables. Journal of Clinical Psychology in Medical Settings.

[ref-26] Hsiao C-Y, Chang C, Chen C-D (2012). An investigation on illness perception and adherence among hypertensive patients. The Kaohsiung Journal of Medical Sciences.

[ref-27] Langenmaier A-M, Amelung VE, Karst M, Krauth C, Püschner F, Urbanski D, Schiessl C, Thoma R, Klasen B (2019). Subgroups in chronic low back pain patients–a step toward cluster-based, tailored treatment in inpatient standard care: on the need for precise targeting of treatment for chronic low back pain. GMS German Medical Science.

[ref-28] Lauritsen J (2007). Danske normtal for Euroqol-5d. http://uag.dk/simpelfunktion/pdf/eq5dknorm.pdf.

[ref-29] Leadley RM, Armstrong N, Reid KJ, Allen A, Misso KV, Kleijnen J (2014). Healthy aging in relation to chronic pain and quality of life in Europe. Pain Practice.

[ref-30] Leventhal EA (1984). Aging and the perception of illness. Research on Aging.

[ref-31] Leventhal H, Benyamini Y, Brownlee S, Diefenbach M, Leventhal EA, Patrick-Miller L, Robitaille C (1997). Illness representations: theoretical foundations. Perceptions of Health and Illness.

[ref-32] Løchting I, Garratt A, Storheim K, Werner E, Grotle M (2013). Evaluation of the brief illness perception questionnaire in sub-acute and chronic low back pain patients: data quality, reliability and validity. Journal of Pain and Relief.

[ref-33] Ludwig C, Luthy C, Allaz A-F, Herrmann F, Cedraschi C (2018). The impact of low back pain on health-related quality of life in old age: results from a survey of a large sample of Swiss elders living in the community. European Spine Journal.

[ref-34] Mailloux J, Finno M, Rainville J (2006). Long-term exercise adherence in the elderly with chronic low back pain. American Journal of Physical Medicine & Rehabilitation.

[ref-35] Morton L, De Bruin M, Macfarlane GJ (2021). Illness perceptions and illness behaviours in back pain: a cross-sectional cluster analysis. European Journal of Pain.

[ref-36] Moss-Morris R, Weinman J, Petrie K, Horne R, Cameron L, Buick D (2002). The revised illness perception questionnaire (IPQ-R). Psychology and Health.

[ref-37] Overholser BR, Sowinski KM (2008). Biostatistics primer: part 2. Nutrition in Clinical Practice.

[ref-38] Patel RP, Taylor SD (2002). Factors affecting medication adherence in hypertensive patients. Annals of Pharmacotherapy.

[ref-39] Qaseem A, Wilt TJ, McLean RM, Forciea MA (2017a). Noninvasive treatments for acute, subacute, and chronic low back pain: a clinical practice guideline from the American College of Physicians. Annals of Internal Medicine.

[ref-40] Qaseem A, Wilt TJ, McLean RM, Forciea MA, Clinical Guidelines Committee of the American College of Physicians (2017b). Noninvasive treatments for acute, subacute, and chronic low back pain: a clinical practice guideline from the american college of physicians. Annals of Internal Medicine.

[ref-41] Rabin R, Charro F de (2001). EQ-5D: a measure of health status from the EuroQol Group. Annals of Medicine.

[ref-42] Sørensen J, Davidsen M, Gudex C, Pedersen KM, Brønnum-Hansen H (2009). Danish EQ-5D population norms. Scandinavian Journal of Public Health.

[ref-43] Thomas E, Peat G, Harris L, Wilkie R, Croft PR (2004). The prevalence of pain and pain interference in a general population of older adults: cross-sectional findings from the North Staffordshire Osteoarthritis Project (NorStOP). Pain.

[ref-44] Ünal Ö, Akyol Y, Tander B, Ulus Y, Terzi Y, Kuru Ö (2019). The relationship of illness perceptions with demographic features, pain severity, functional capacity, disability, depression, and quality of life in patients with chronic low back pain. Turkish Journal of Physical Medicine and Rehabilitation.

[ref-45] Van Middelkoop M, Rubinstein SM, Kuijpers T, Verhagen AP, Ostelo R, Koes BW, Van Tulder MW (2011). A systematic review on the effectiveness of physical and rehabilitation interventions for chronic non-specific low back pain. European Spine Journal.

[ref-46] Van Oort L, Schröder C, French D (2011). What do people think about when they answer the brief illness perception questionnaire? A ‘think-aloud’ study. British Journal of Health Psychology.

[ref-47] Viniol A, Jegan N, Hirsch O, Leonhardt C, Brugger M, Strauch K, Barth J, Baum E, Becker A (2013). Chronic low back pain patient groups in primary care–a cross sectional cluster analysis. BMC Musculoskeletal Disorders.

[ref-48] Vos T, Abajobir AA, Abate KH, Abbafati C, Abbas KM, Abd-Allah F, Abdulkader RS, Abdulle AM, Abebo TA, Abera SF (2017). Global, regional, and national incidence, prevalence, and years lived with disability for 328 diseases and injuries for 195 countries, 1990–2016: a systematic analysis for the Global Burden of Disease Study 2016. The Lancet.

[ref-49] Willis GB (2004). Cognitive interviewing: a tool for improving questionnaire design.

[ref-50] Windgassen S, Moss-Morris R, Goldsmith K, Chalder T (2018). The importance of cluster analysis for enhancing clinical practice: an example from irritable bowel syndrome.

[ref-51] Wittrup-Jensen KU, Lauridsen J, Gudex C, Pedersen KM (2009). Generation of a Danish TTO value set for EQ-5D health states. Scandinavian Journal of Public Health.

