# Supplementary material for: The correlation between illness perception, pain intensity and quality of life in elderly with low back pain in Denmark: a cross-sectional study
Source: PeerJ. 2022 Oct 14;10:e14129. doi: 10.7717/peerj.14129 (PMC9575667; doi:10.7717/peerj.14129)
Supplement: Supplemental Information 2 [file peerj-10-14129-s002.pdf]

STROBE Statement—Checklist of items that should be included in reports of ***cross-sectional studies***

	Item No	Recommendation
**Title and abstract**	1	(*a*) Indicate the study's design with a commonly used term in the title or the abstract Abstract page 2
		(*b*) Provide in the abstract an informative and balanced summary of what was done and what was found Abstract page 2
**Introduction**		
Background/rationale	2	Explain the scientific background and rationale for the investigation being reported page 3-4
Objectives	3	State specific objectives, including any prespecified hypotheses Background and hypotheses section page 4
**Methods**		
Study design	4	Present key elements of study design early in the paper Materials & Methods section page 5
Setting	5	Describe the setting, locations, and relevant dates, including periods of recruitment, exposure, follow-up, and data collection Method section page 5
Participants	6	(*a*) Give the eligibility criteria, and the sources and methods of selection of participants Method (Participant) section page 5
Variables	7	Clearly define all outcomes, exposures, predictors, potential confounders, and effect modifiers. Give diagnostic criteria, if applicable Variables and outcome measures section page 5-8
Data sources/ measurement	8*	For each variable of interest, give sources of data and details of methods of assessment (measurement). Describe comparability of assessment methods if there is more than one group Variables and outcome measures section page 5-8
Bias	9	Describe any efforts to address potential sources of bias Materials & Methods section page 4-5
Study size	10	Explain how the study size was arrived at Materials & Methods (Participant) section page 4 and variables and outcome measures page 5
Quantitative variables	11	Explain how quantitative variables were handled in the analyses. If applicable, describe which groupings were chosen and why Variables and outcome measures section page 6-7 and Statistical analyses section page 8.
Statistical methods	12	(*a*) Describe all statistical methods, including those used to control for confounding page 8
		(*b*) Describe any methods used to examine subgroups and interactions Statistical analyses section page 8
		(*c*) Explain how missing data were addressed Results section page 9
		(*d*) If applicable, describe analytical methods taking account of sampling strategy Statistical analyses section page 8
		(*e*) Describe any sensitivity analyses n/a
**Results**		
Participants	13*	(a) Report numbers of individuals at each stage of study—eg numbers potentially eligible, examined for eligibility, confirmed eligible, included in the study, completing follow-up, and analysed Results section page 9-10 and Fig. 1 flowchart
		(b) Give reasons for non-participation at each stage Flowchart Fig. 1
		(c) Consider use of a flow diagram Done
Descriptive data	14*	(a) Give characteristics of study participants (eg demographic, clinical, social) and

		information on exposures and potential confounders Table 1 and results section page 9-10
		(b) Indicate number of participants with missing data for each variable of interest Table 1 and 3
Outcome data	15*	Report numbers of outcome events or summary measures Table 1 and 3 and results section page 9-12
Main results	16	(*a*) Give unadjusted estimates and, if applicable, confounder-adjusted estimates and their precision (eg, 95% confidence interval). Make clear which confounders were adjusted for and why they were included Table 1, 2, 3 and figure 2 and results section page 9-12 (only unadjusted estimates)
		(*b*) Report category boundaries when continuous variables were categorized n.a.
		(*c*) If relevant, consider translating estimates of relative risk into absolute risk for a meaningful time period n.a.
Other analyses	17	Report other analyses done—eg analyses of subgroups and interactions, and sensitivity analyses n.a.
**Discussion**		
Key results	18	Summarise key results with reference to study objectives Discussion page 13-14
Limitations	19	Discuss limitations of the study, taking into account sources of potential bias or imprecision. Discuss both direction and magnitude of any potential bias Discussion page 14
Interpretation	20	Give a cautious overall interpretation of results considering objectives, limitations, multiplicity of analyses, results from similar studies, and other relevant evidence Discussion page 13 and Conclusion page 14
Generalisability	21	Discuss the generalisability (external validity) of the study results Discussion page 14
**Other information**		
Funding	22	Give the source of funding and the role of the funders for the present study and, if applicable, for the original study on which the present article is based Done

*Give information separately for exposed and unexposed groups.

Note: An Explanation and Elaboration article discusses each checklist item and gives methodological background and published examples of transparent reporting. The STROBE checklist is best used in conjunction with this article (freely available on the Web sites of PLoS Medicine at http://www.plosmedicine.org/, Annals of Internal Medicine at http://www.annals.org/, and Epidemiology at http://www.epidem.com/). Information on the STROBE Initiative is available at www.strobe-statement.org.
